# Impact of Meningococcal ACWY Vaccination Program during 2017–18 Epidemic, Western Australia, Australia

**DOI:** 10.3201/eid3002.230144

**Published:** 2024-02

**Authors:** Krist Ewe, Parveen Fathima, Paul Effler, Carolien Giele, Peter Richmond

**Affiliations:** Wesfarmers Centre of Vaccines and Infectious Diseases, Perth, Western Australia, Australia (K. Ewe, P. Fathima, P. Richmond);; Perth Children’s Hospital, Perth (K. Ewe, P. Richmond);; Sydney School of Public Health, University of Sydney, Sydney, New South Wales, Australia (P. Fathima);; Communicable Disease Control Directorate, Western Australia Department of Health, Perth (P. Effler, C. Giele);; University of Western Australia School of Medicine, Perth (P. Richmond).

**Keywords:** meningitis/encephalitis, bacteria, invasive meningococcal disease, vaccination program, MenACWY, quadrivalent meningococcal vaccine, Australia

## Abstract

The rising incidence of invasive meningococcal disease (IMD) caused by *Neisseria meningitidis* serogroup W in Western Australia, Australia, presents challenges for prevention. We assessed the effects of a quadrivalent meningococcal vaccination program using 2012–2020 IMD notification data. Notification rates peaked at 1.8/100,000 population in 2017; rates among Aboriginal and Torres Strait Islander populations were 7 times higher than for other populations. Serogroup W disease exhibited atypical manifestations and increased severity. Of 216 cases, 20 IMD-related deaths occurred; most (19/20) were in unvaccinated persons. After the 2017–2018 targeted vaccination program, notification rates decreased from 1.6/100,000 population in 2018 to 0.9/100,000 population in 2019 and continued to decline in 2020. Vaccine effectiveness (in the 1–4 years age group) using the screening method was 93.6% (95% CI 50.1%–99.2%) in 2018 and 92.5% (95% CI 28.2%–99.2%) in 2019. Strategic planning and prompt implementation of targeted vaccination programs effectively reduce IMD.

Invasive meningococcal disease (IMD) remains a public health concern worldwide. The causative organism, *Neisseria meningitidis*, is differentiated into 12 distinct serogroups, of which A, B, C, W, X, and Y are most commonly associated with IMD (*1*). *N. meningitidis* is present in the nasopharynx of nearly 10% of the population without causing disease, and IMD develops in only a small proportion of those persons. IMD is characterized by sudden onset of symptoms (including stiff neck, headache, photophobia, and a characteristic spotty red-purple rash) and rapid clinical progression leading to septicemia or meningitis. IMD-associated mortality is ≈10%–15%; however, more than one third of all IMD patients experience notable long-term or permanent effects, such as skin necrosis, deafness, seizures, or other neurologic sequelae (*2*,*3*). Infants <1 year of age have the highest risk for IMD, followed by a smaller second peak in adolescents and young adults, reflecting the social behavior that increases the nasopharyngeal carriage of meningococcus.

The evolving and unpredictable epidemiology of IMD poses additional challenges to its prevention. *N. meningitidis* serogroups causing IMD are known to change over time. After meningococcal C vaccine was introduced into the Australian National Immunization Program in 2003, IMD notification rates declined from 3.5/100,000 population in 2002 to 0.6/100,000 in 2013; *N. meningitidis* serogroup B (MenB) was responsible for most cases. After 2013, IMD notification rates increased, reaching 1.5/100,000 population in 2017 (*4*). A similar trend was seen in the state of Western Australia (*4*). For many years, MenB was responsible for most IMD notifications in Australia; however, since 2013, the incidence of IMD caused by *N. meningitidis* serogroup W (MenW) has increased (*5*). Globally, MenW IMD is often associated with atypical clinical features, including gastrointestinal symptoms, septic arthritis, pneumonia, and epiglottitis (*6*), along with high rates of illness and death (*7*).

Effective vaccines against *N. meningitidis* serogroups A, B, C, W, and Y are available (*8*). Some countries have included or are considering publicly funding the quadrivalent meningococcal vaccines (ACWY) as part of their national immunization program (*8*). Those decisions are usually influenced by numerous factors, including cost-effectiveness, vaccine efficacy, and effects on public health. In Western Australia, since 2016, the state government has incrementally funded and delivered MenACWY conjugate vaccines through targeted vaccination campaigns in relevant schools, community health centers and immunization clinics. During December 2016–December 2017, the vaccines were offered free to all children 12 months–4 years of age and to teenagers 15–19 years of age in affected and at-risk communities in regional areas (*9*). During October–December 2017, the funded vaccines were extended to persons of all ages in those areas. Beginning in January 2018, the vaccine became available to all children 12 months–4 years of age. During May 2017–March 2019, funding covered students in grades 10–12 and adolescents 15–19 years of age who no longer attended school (Figures 1, 2). We reviewed the effects of the introduction of a targeted quadrivalent meningococcal (ACWY-TT) vaccination program in Western Australia following an outbreak of MenW in 2017–2018.

**Figure 1:**
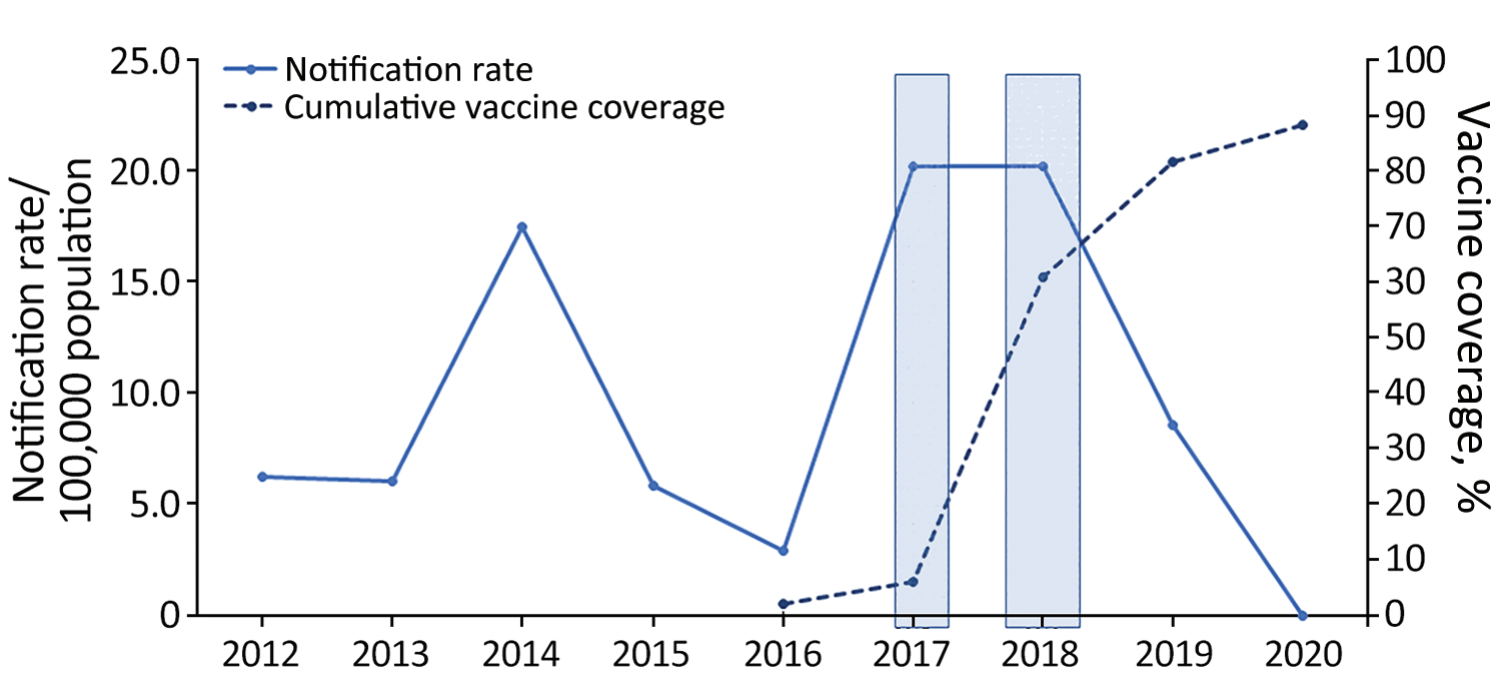
Invasive meningococcal disease notification rates (solid line) and cumulative meningococcal ACWY vaccine coverage rates (dotted line) among children 12 months–4 years of age, Western Australia, 2012–2020. First shaded box indicates the period of December 2016–March 2017, during which the local Western Australia government funded the meningococcal ACWY conjugate vaccine for children 12 months–4 years of age and adolescents 15–19 years of age in selected affected and at-risk regional areas. Second shaded box indicates period of October–December 2017, during which vaccine was funded for people of all ages in selected affected and at-risk regional areas. Beginning in January 2018, the vaccine was funded and available to all children 12 months–4 years of age.

**Figure 2:**
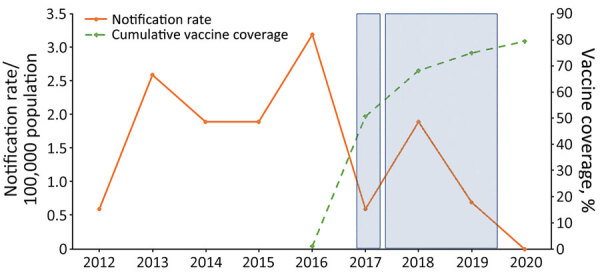
Invasive meningococcal disease notification rates (solid line) and cumulative meningococcal ACWY vaccine coverage rates (dotted line) among adolescents 15–19 years of age, Western Australia, 2012–2020. First shaded box indicates the period of December 2016–March 2017, during which the local Western Australia government funded the meningococcal ACWY conjugate vaccine for children 12 months–4 years and adolescents 15–19 years in selected affected and at-risk regional areas. Second shaded box indicates period of May 2017–March 2019, during which vaccine was funded for students in grades 10–12 and adolescents 15–19 years of age no longer enrolled in school.

## Methods

The Communicable Disease Control Directorate at the Western Australia Department of Health conducts enhanced surveillance for IMD statewide. All pathology laboratories within the state routinely notify the Directorate of any laboratory-confirmed or probable (suspected) diagnosis of IMD. A confirmed case is defined as one in which *N. meningitidis* is identified by standard microbiological methods from a normally sterile site (isolation of *N. meningitidis* by culture or detection of *N. meningitidis* DNA by nucleic acid amplification testing). A probable case is illness in a person experiencing clinical manifestations consistent with IMD but not confirmed by standard microbiological methods. Case data collected routinely include medical history, gender, region of residence at the time of disease onset, vaccination status, risk factors, indigenous status, clinical manifestations, serogroup information, and outcome. The data are captured on the Western Australia Notifiable Infectious Diseases Database. For this study, we extracted deidentified data on all IMD notifications in Western Australia with a date of onset during 2012–2020.

Cases were categorized into age groups of 0–4 years, 5–9 years, 10–14 years, 15–19 years, 20–24 years, 25–44 years, 45–64 years, and >65 years. Using annual population estimates (obtained from Rates Calculator, Epidemiology Branch, Western Australia Department of Health), we calculated age-specific and age-standardized notification rates by Aboriginal and Torres Strait Islander (hereafter referred to as Aboriginal) status. We analyzed clinical manifestations and outcomes by causative serogroup for laboratory-confirmed IMD cases. We calculated case-fatality rates (CFRs) according to year and serogroup.

We used a hierarchy of clinical manifestations to assign a single clinical syndrome category. We assigned *N. meningitidis* detected or isolated from a single normally sterile site to that site. *N. meningitidis* detected or isolated from 2 normally sterile sites was assigned the site of isolation other than blood. For example, if *N. meningitidis* was detected in blood and cerebrospinal fluid, then meningitis would be assigned. *N. meningitidis* was not isolated from >3 sites in any of the cases in this study.

We collected patient or parental recall regarding previous receipt of meningococcal vaccinations for all IMD notifications. Where available, we validated data on vaccine uptake using the Australian Immunization Register (*10*). We reported vaccination status of persons with IMD as fully vaccinated (received all recommended doses of the meningococcal vaccine according to the state or regionally funded vaccination program), not vaccinated (vaccine available, but not received), unknown (vaccination status not known or documented) or nonapplicable (vaccine not publicly available or funded for the relevant groups). IMD notification rates were assessed against data on MenACWY vaccination coverage; we compared rates before and after the introduction of the target meningococcal vaccination program in Western Australia.

**Figure 7:**
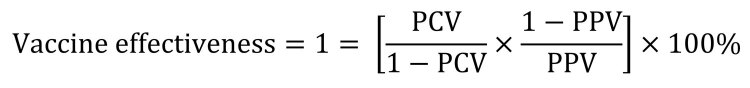
Vaccine effectiveness formula.

Using the screening method (*11*), we estimated the effectiveness of meningococcal vaccine against notified IMD for the vaccine-eligible age-groups (i.e., 1–4 years, 5–14 years and 15–19 years) using the following formula (Figure 7):

where PCV is the proportion of IMD case-patients who were vaccinated and PPV is the proportion of population vaccinated.

## Results

**Figure 3:**
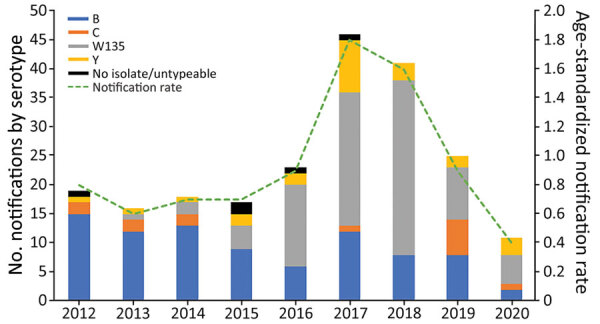
Invasive meningococcal disease notifications (N = 216) and age-standardized notification rates (per 100,000 population), by year and serogroup, Western Australia, 2012–2020.

During January 2012–December 2020, a total of 216 cases of IMD were reported; 113 (52%) patients were male and 103 (48%) female. Of the 216 cases, 213 (98.6%) were laboratory-confirmed cases, and the remainder (n = 3) were diagnosed on the basis of a high index of clinical suspicion. Sixty-one percent of cases (131/216) occurred in residents of the Perth Metropolitan area, home to ≈80% of the Western Australia population (*12*). The age-standardized IMD notification rate rose from a baseline of 0.8/100,000 population (n = 19) in 2012 to a peak of 1.8/100,000 (n = 46) in 2017, then declined to 0.9/100,000 in 2019, an incidence rate ratio of 0.53 (CI 0.31–0.88; p = 0.011). In 2020, the IMD notification rate declined further to 0.4/100,000 population (Figure 3).

MenB accounted for 79% (n = 15/19) of all IMD cases in 2012, in contrast to 2 cases of MenC, 1 case of MenY, and 0 cases of MenW in the same year (the remaining case was nongroupable). The proportion of IMD notifications caused by MenB waned over the years, but the number of IMD cases caused by MenW gradually increased (Figure 3); MenW overtook MenB as the dominant serogroup in 2016, accounting for 61% (n = 14/23) of all IMD notifications in Western Australia.

**Figure 4:**
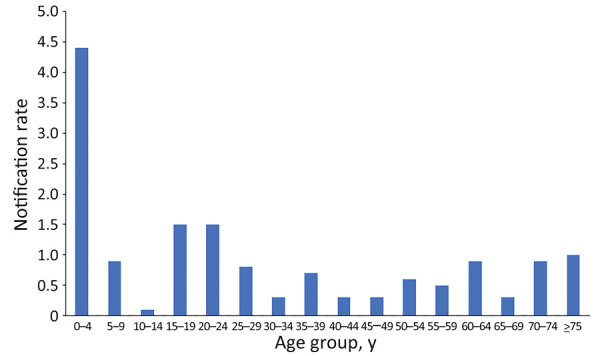
Invasive meningococcal disease notification rates (per 100,000 population), by age group, Western Australia, 2012–2020.

**Figure 5:**
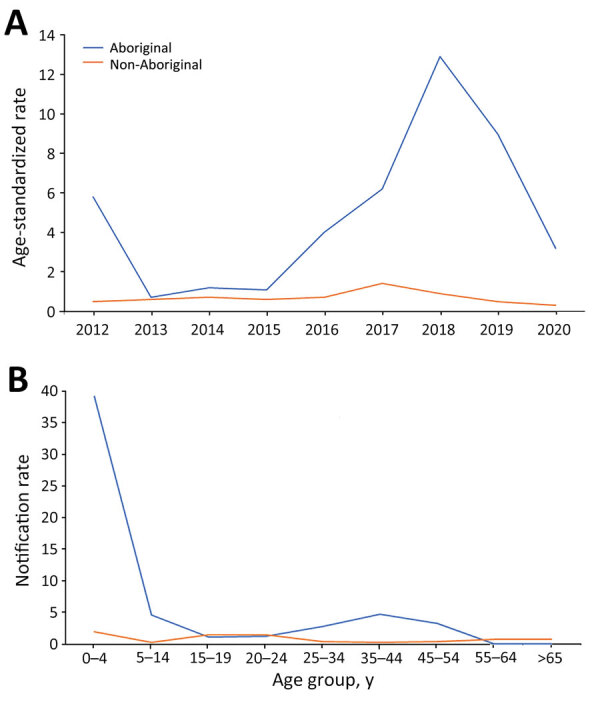
Invasive meningococcal disease notification rates (per 100,000 population) by population group in Western Australia (Aboriginal vs. non-Aboriginal), 2012–2020, by year (A) and age group (B).

As expected, the highest IMD notification rates overall during 2012–2020 were among children 0–4 years of age (4.4/100,000 notifications) (Figure 4), followed by a second, smaller peak in adolescents 15–19 years and young adults 20–24 years (1.5/100,000 notifications). Notification rates were lower in other age groups, rising slightly for those >60 years of age. In total, 63/216 cases (29.2%) of IMD occurred among the Aboriginal population. The overall (2012–2020) age-standardized notification rate for IMD among the Aboriginal population (4.9/100,000 population) was 7 times higher than in the non-Aboriginal population (0.7/100,000 population). This difference was largely because of an IMD outbreak among the Aboriginal population in 2017–2019, beginning in Central Australia and spreading to neighboring states; 65% (n = 41/63) of IMD cases in the Aboriginal population occurred during this period, and most (63%; n = 26) occurred in children <5 years of age. Overall, 76.2% (n = 48) of all IMD notifications among Aboriginal persons occurred in the 0–9 years age group, and most of those were in the 0–4 years age group (n = 40) (Figure 5).

**Figure 6:**
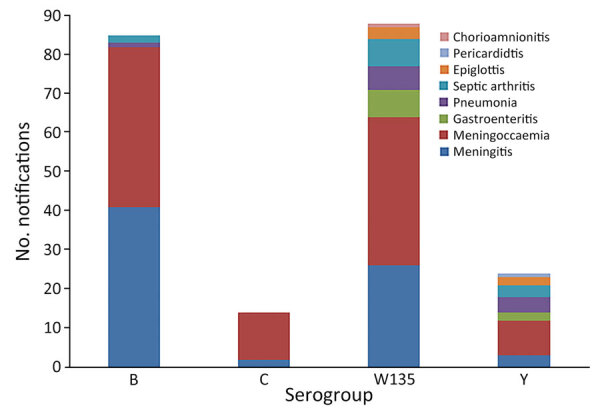
Clinical manifestations of invasive meningococcal disease by serogroup, Western Australia, 2012–2020. Categories of clinical manifestation include only meningococcal isolates that were typable.

The overall clinical manifestations ranged from typical septicemia (47.4%; n = 100/211) and meningitis (34.1%; n = 72/211) to more atypical symptoms, such as septic arthritis (6%), pneumonia (5%), epiglottitis/pharyngitis (2%), pericarditis (0.5%), and chorioamnionitis (0.5%) (Figure 6). Our study showed that 27% (24/88) of MenW case-patients and 50% (12/24) of MenY case-patients displayed atypical symptoms. The overall CFR was 9.3% (n = 20); MenW was identified in most IMD-related deaths (60%; n = 12/20), followed by MenB (35%; n = 7). With the exception of 1 case-patient whose meningococcal vaccination status was unknown, the rest of the IMD-related deaths (19/20) were in persons who were not vaccinated.

Overall, rates of meningococcal ACWY vaccine uptake were high in the targeted groups (the vaccine publicly funded for persons 12 months–4 years, 15–19 years, and for all ages in select affected or at-risk regional areas). Since the introduction of the quadrivalent meningococcal ACWY (Nimenrix) vaccination program in December 2016, uptake rates among children 1–4 years increased rapidly, reaching 90% of all children in this age group by 2020 (Figure 1). A similar trend was noted in the 15–19-year age group, in which rates reached 80% by 2020 (Figure 2). The incidence of IMD declined from 1.6/100,000 population in 2018 to 0.9/100,000 population in 2019 (corresponding to 30 cases in 2018 and 9 cases in 2019) after the introduction of the targeted vaccination program. In 2020, no notifications of IMD caused by serogroups A, C, W and Y in the age cohorts targeted for vaccination were recorded.

In the highest-risk age group (12 months–4 years), the vaccine effectiveness calculated using the screening method was 93.6% (95% CI 50.1%–99.2%) for 2018 and 92.5% (95% CI 28.2%–99.2%) for 2019. Power was inadequate to generate a meaningful interpretable value for the 5–14- and 15–19-year age groups for whom the vaccine was available.

## Discussion

IMD incidence data show a change in the epidemiology of IMD in Western Australia from 2012 to 2020. The dominance of MenB as the primary cause of IMD was succeeded by a rise in the number of notifications of IMD caused by MenW and, to a lesser extent, MenY. The rise in MenW notifications in Western Australia and Australia as a whole is not an isolated occurrence. Over the past 2 decades, increases in the incidence of IMD caused by MenW have been reported in other settings (*3*,*13*–*20*).

In Western Australia, the rise in IMD notifications eventually led to an IMD outbreak in 2017 and 2018, disproportionately affecting Aboriginal populations. Elevated rates of IMD have been described previously among Aboriginal Australian populations (*21*–*23*) and in other specific populations outside Australia, such as African American persons (*24*) and Pacific Islanders (*25*). Up to 1 in 5 Aboriginal Australians live in remote or very remote areas (*26*). A similar proportion of Aboriginal Australians live in overcrowded households and are more likely to live in suboptimal conditions, such as having limited access to essential services and sanitation (*27*). The higher incidence rates of IMD disease in Aboriginal populations in Australia have been attributed to specific risk factors, whereas higher mortality rates are thought to be linked to limited timely access to healthcare services. Younger Aboriginal children experience higher rates of IMD than the general population, potentially because of a combination of risk factors, such as the immaturity of their immune system, frequent viral upper respiratory tract infections, exposure to passive smoking, household crowding (*28*), and lower vaccination rates (*29*).

Atypical clinical manifestations and higher CFR in our cohort were seen more frequently with IMD caused by MenW and MenY than for other serogroups, consistent with findings from other countries where severe cases were caused by a hypervirulent strain belonging to the sequence type 11 clonal complex (cc11) (*20*,*30*). The 2017 Australian Meningococcal Surveillance Programme reported that, among the MenW strains that were able to be genotyped, 59% (74/125) were sequence type 11, the same strain circulating in the United Kingdom and South America since 2009 (*31*). Advances in sequencing technology have enabled further characterization of this clonal complex into distinct lineages and sublineages, revealing an evolution of genetically (and geo-temporally) diverse global cc11 populations that exhibit different epidemiologic properties (*32*).

Although the hypervirulent MenW cc11 strains have been shown to diverge from a MenC cc11 ancestral strain by capsular switching (*33*,*34*), some appear unrelated to the contemporary MenC cc11 strains (*35*). Furthermore, certain variants, particularly the cc11/non–ET-15 variants, lack the virulence factors present in cc11/ET-15 strains associated with more aggressive infections in the cc11/ET-15 strains (*36*). In our cohort, IMD caused by MenW was also associated with a high mortality rate, consistent with a systematic review and metaanalysis of CFR of IMD (*37*).

The clinical manifestations of IMD caused by MenY are less well characterized, but a retrospective observational study performed on a large cohort in Sweden reported atypical symptoms, such as pneumonia (19%) and septic arthritis (10%), particularly in older patients (*38*). The implications of the rise of IMD caused by MenW and MenY ultimately manifesting with more atypical symptoms might lead to delayed diagnosis or misdiagnosis even by experienced clinicians and to use of antibiotics that are ineffective against *N. meningitidis*, leading to rapid progression of disease and death.

Vaccination remains the most effective strategy for preventing IMD and its complications. The 2017–18 outbreak of MenW, and to a lesser extent MenY, in Western Australia led to the rapid implementation of a targeted vaccination program with the quadrivalent meningococcal ACWY vaccine (Nimenrix) in December 2016, starting with high-risk groups in communities located in geographic areas with increased IMD incidence.

Success stories abound of meningococcal vaccine programs responding to IMD outbreaks across the globe. In 2004, the introduction of mass vaccinations with the outer membrane vesicle vaccine against the serogroup B epidemic strain in New Zealand (MeNZB) reduced the risk for infection by 4-fold, and by 2009 the incidence rate of IMD had declined to 3.3/100,00 population, compared with 17.4/100,000 population at its peak (*39*). Similarly, the use of the recombinant meningococcal serogroup B vaccine in the United States (4CMenB) in response to a university campus outbreak saw no further cases linked to the university after the initiation of the vaccine program (*40*). More impressive was the prophylactic use of the MenA conjugate vaccine in Burkina Faso, Mali, and Niger (MenAfriVac). A 10-day vaccination campaign in Burkina Faso saw ≈11 million residents get vaccinated (*41*), 3 million residents were vaccinated in Niger in 10 days (*42*), and a 14-day campaign in Mali saw 4.5 million residents get vaccinated. The success of that vaccination campaign cannot be understated; 5 years after the mass vaccination campaign, Burkina Faso had gone from a hyperendemic state to only recording sporadic cases of IMD caused by MenA (*43*). In a separate targeted vaccination program, the introduction of a 2-dose 4CMenB infant schedule as part of a publicly funded UK immunization program resulted in a 50% reduction in the incidence of MenB cases among the vaccine-eligible cohort within 10 months (*44*).

Our data show that IMD is uncommon among those who are fully vaccinated, and fatal cases occur primarily in those who are not vaccinated. The rapid implementation of a targeted vaccination program in 2017–2018 in young children most at risk (those 1–4 years of age), as well as in adolescents and young adults (15–24 years) who have the highest carriage rates, led to a dramatic reduction in the number of notifications for Western Australia in 2019 that continued into 2020. The rapid and high vaccination uptake rate reflects effective public health communication and strong awareness of the outbreak among the Aboriginal population in remote or socially disadvantaged areas, which traditionally have lower vaccination uptake than the general population (*45*). The absence of cases in the vaccinated cohort supports the notion that vaccination is effective in preventing disease and death.

Lockdowns related to COVID-19 in 2020 were thought to affect the reduction in the number of IMD notifications in that year. A study from France showed that the overall number of IMD notifications, particularly of hyperinvasive strains, reduced during the lockdown period during January 2020–May 2020, but the proportion of cases associated with respiratory symptoms (MenY) increased during that period (*46*). Analysis of surveillance data submitted from laboratories in 26 countries and territories across 6 continents demonstrated a substantial reduction of invasive diseases caused by respiratory pathogens, including *N. meningitidis*, during the same lockdown period, likely because of strict containment policies (*47*). As part of the national response to the COVID-19 pandemic in Western Australia, international borders were closed to nonresidents on March 20, 2020, and subsequent restrictions were placed on interstate travel on April 6, 2020. A statewide stay-at-home restriction, including an extended school holiday, was imposed from March 27 through April 27, 2020; schools resumed at near capacity after that. Those restrictions could arguably have affected opportunities for exposure to *N. meningitidis.* Of note, however, a substantive reduction in the number of IMD notifications was already evident in 2019, and the effect persisted throughout 2020. The statewide restrictions on interstate travel and Western Australia school closures were thought to be too brief to have any long-term effect on transmission of *N. meningitidis*, and, in our opinion, increases in handwashing and surface cleaning and physical distancing measures would not likely fully explain the absence of IMD cases caused by serogroups A, C, W, and Y among persons 0–4 years of age and adolescents in 2020.

In conclusion, the epidemiology of IMD is constantly changing. Both direct and herd protection are essential because of the atypical manifestation and high mortality associated with some serogroups, which leads to delayed diagnosis and potentially increased case fatality. The vaccine effectiveness was high in the most at-risk group using the screening method. Rapidly implementing meningococcal vaccination programs with high coverage in at-risk populations is effective in reducing the incidence of IMD in an outbreak setting. Vaccination against meningococcal disease, particularly MenW and MenY, should continue to be encouraged.
